# Lizards on Ice: Evidence for Multiple Refugia in *Liolaemus pictus* (Liolaemidae) during the Last Glacial Maximum in the Southern Andean Beech Forests

**DOI:** 10.1371/journal.pone.0048358

**Published:** 2012-11-27

**Authors:** Iván Vera-Escalona, Guillermo D'Elía, Nicolás Gouin, Frank M. Fontanella, Carla Muñoz-Mendoza, Jack W. Sites, Pedro F. Victoriano

**Affiliations:** 1 Departamento de Zoología, Facultad de Ciencias Naturales y Oceanográficas, Universidad de Concepción, Concepción, Chile; 2 Instituto de Ciencias Ambientales y Evolutivas, Universidad Austral de Chile, Valdivia, Chile; 3 Centro de Estudios Avanzados en Zonas Áridas, Facultad de Ciencias del Mar, Universidad Católica del Norte, Coquimbo, Chile; 4 Department of Biology and Bean Life Science Museum, Brigham Young University, Provo, Utah, United States of America; 5 Centro de Investigaciones de Ecosistemas de la Patagonia CIEP, Coihaique, Chile; Fordham University, United States of America

## Abstract

Historical climate changes and orogenesis are two important factors that have shaped intraspecific biodiversity patterns worldwide. Although southern South America has experienced such complex events, there is a paucity of studies examining the effects on intraspecific diversification in this part of the world. *Liolaemus pictus* is the southernmost distributed lizard in the Chilean temperate forest, whose genetic structure has likely been influenced by Pleistocene glaciations. We conducted a phylogeographic study of *L. pictus* in Chile and Argentina based on one mitochondrial and two nuclear genes recovering two strongly divergent groups, Northern and Southern clades. The first group is distributed from the northernmost limit of the species to the Araucanía region while the second group is distributed throughout the Andes and the Chiloé archipelago in Southern Chile. Our results suggest that *L. pictus* originated 751 Kya, with divergence between the two clades occurring in the late Pleistocene. Demographic reconstructions for the Northern and Southern clades indicate a decrease in effective population sizes likely associated with Pleistocene glaciations. Surprisingly, patterns of genetic variation, clades age and historical gene flow in populations distributed within the limits of the Last Glacial Maximum (LGM) are not explained by recent colonization. We propose an “intra-Andean multiple refuge” hypothesis, along with the classical refuge hypothesis previously proposed for the biota of the Chilean Coastal range and Eastern Andean Cordillera. Our hypothesis is supported by niche modelling analysis suggesting the persistence of fragments of suitable habitat for the species within the limits of the LGM ice shield. This type of refuge hypothesis is proposed for the first time for an ectothermic species.

## Introduction

The landscape of southern South America has been shaped by several climatic and geological processes, of which two are especially important because of the magnitude of their evolutionary consequences: orogenic changes associated to the uplift of Andes [1] and glacial cycles with alternate levels of contrasting temperatures and concordant expansion and retreat of ice shields during the Pleistocene-Holocene period [2]. Andean uplift and subsequent interaction with the Pacific wet wind drift sustains the unique Valdivian Forest, characterized by a high level of endemism throughout a narrow band (∼150–250 km wide) on the western Andes in Chile, from 36°S to 56°S, and adjacent Argentinean areas [3], [4]. These forests, dominated by *Nothofagus* tree species and their associated communities, are formally recognized as a Chilean Biodiversity Hot Spot [5].

**Figure 1:**
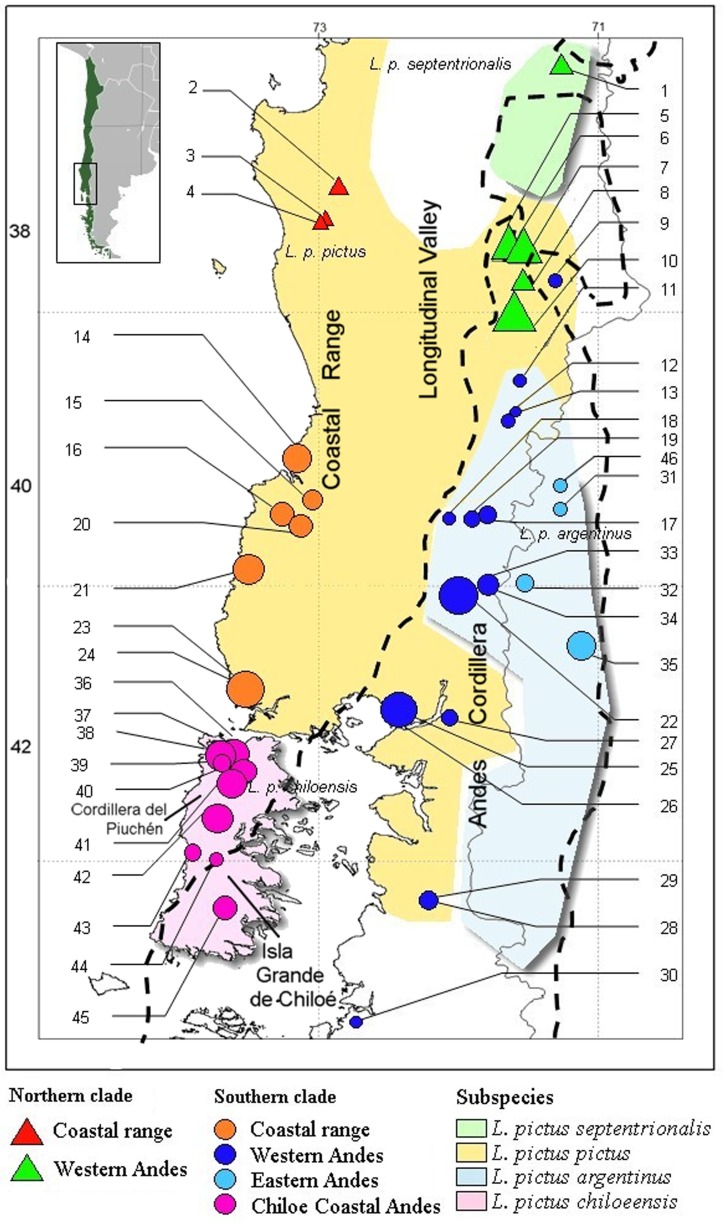
Sample sites (as detailed in Table S1), and subspecies distributions of *L. pictus*. Triangles and circles show the Northern and Southern clades, respectively. Symbols sizes are proportional to sample sizes, and dashed line is the estimated limit of the ice shield during LGM according to Heusser [11].

The Pleistocene epoch was characterized by several global glacial cycles that deeply impacted polar and temperate regions. The well-studied Last Glacial Maximum (LGM), which occurred approximately 23,000–18,000 years ago (ya) [6], modified landscapes throughout the distribution of the Southern Andean temperate forest due to the presence of an ice shield that extended from ∼36°S to ∼42°S. At maximum extent, this ice shield lowered sea level by ∼120 m, exposing coastal areas and connecting near shore islands with the mainland. Glaciers likely covered all land south of the Cordillera del Piuchén on Chiloé Island (Fig. 1; [7], [8], [9]), but this ice shield could have presented small ice-free “islands”, mainly in the coastal range, small Andean valleys, and active volcanic zones. For example, both the Ñuble region (∼37°S) and the Lonquimay Valley (∼38°S) in the Chilean Andes were likely covered by discontinuous ice shields [10], [11], [12], which may explain present day patterns of genetic variation in several organisms such as freshwater crabs and frogs [13], [14]. Recent studies have presented evidence for fragmented intra-ice shield refugia and small populations of plants and poikilothermic vertebrates that persisted through cyclic glaciations [13], [14], [15]. The phylogeographic consequences of these glacial phases would include: (1) fragmentation and decrease in population effective sizes of widely distributed species during phases of ice shield spread, with this effect amplified towards higher latitudes, and (2) evidence for subsequent postglacial range expansions, again with stronger signatures at southern-most latitudes. A contrasting pattern should also be evident in the presumably less affected populations from Central Chile, mainly along the Pacific coast in the Coastal Cordillera. Indeed, these areas are thought to have been relatively stable [7], [8], [9], [16] and therefore we should expect stronger signals of demographic equilibrium with increasing northern latitudes.

To date there is no strong evidence for intraglacial refugia throughout the Andes for heterothermic species. During the LGM, direct and periglacial effects may have been stronger for poikilothermic than for homeothermic species because lower temperatures would exclude the former from broad areas [17], [18]. One group of terrestrial endemic heterothermic vertebrates that fits this situation is the pool of lizard species of the genus *Liolaemus*. This clade is endemic to temperate South America and includes approximately 240 described species, of which 70 occur in Chile [19]. One of the most conspicuous species of this genus is *Liolaemus pictus* [20], a medium-sized viviparous lizard inhabiting *Nothofagus* forests [21]. It is the southernmost West-Andean *Liolaemus* species [22] and one of the few lizards adapted to live in cold, rainy woodland habitats. Ecologically *L. pictus* ranges from sea level to 1600 m, along over 800 km. Its western distribution covers Chile from the Región del Maule (∼36°S) to the Región de Los Lagos including some islands in the Chiloé archipelago (43°S) while its eastern distribution covers part of the Northern Argentinean Patagonia [23] (Fig. 1). This species shows extensive geographic variation in color and six subspecies have been described, including three mainland forms (Fig. 1), while three others are on near shore islands [24]. Color based subspecies occur in sympatry at some localities, suggesting the possibility of divergence in isolation followed by dispersal and secondary contact [25], [26], [27], [28]. However, the fact that different color patterns are representative of a polymorphic feature has not been ruled out.

Previous studies on some *Liolaemus* species have shown no significant reduction of genetic variation, effective population size (N_e_) and distributional ranges in areas far from the LGM effects. In contrast, southern populations and those near the Andes exposed to direct and periglacial effects do show signatures of range reductions and loss of genetic variation [28], [29]. Vidal et al. [24] suggested for *L. pictus* that during the LGM some mainland localities could have acted as refugia. Remarkably, none of the *Liolaemus* studies examined the timing of the inferred demographic changes, either retractions or expansions, putatively associated to the Quaternary glaciations. These scenarios may be further tested by assessing the congruence between past stability areas inferred by using ecological niche modeling [30], [31], [32] and the demographic inferences gathered from the assessment of genetic variation and evolutionary relationships [31], [33].

The objective of this study was therefore to assess the phylogeographic structure of *L. pictus,* based on dense geographic sampling, niche modeling, and genetic information from one cytoplasmic and two nuclear loci, in order to test two hypotheses. First, we predict a pattern of stronger influences of recent glaciations (LGM), both in populations in the eastern (Andean) and southern distribution of this species. If so, we should expect to see signals of disequilibrium between mutation and genetic drift, with a predominance of derived haplotypes in glaciated and recolonized areas, evidence of reductions in effective population size (Ne) during the LGM, and increases in Ne during glacial retreat. In addition, we hypothesize that if the effects of the glaciations in the Andes and within the large ice sheet limits were strong, most of population migrations during a glacial retreats would have been directional from the western refugia towards the Andes. Because phylogeographic histories recovered from mtDNA sequences alone might be discordant with nuclear gene histories [34], [35], [36], we analyzed both in this study. The geographic patterns of evolutionary relationships among populations, as well as the distribution of genetic variation, were compared with the past and present distributions of potential habitat for *L. pictus*. We also tested the congruence between the inferred evolutionary lineages and the patterns of genetic differentiation throughout their distributions, and discussed these in the context of the recognized subspecies of *L. pictus*.

## Materials and Methods

### Sample collection

We analyzed 196 specimens of *L. pictus* collected at 46 localities across the entire latitudinal range of the species, between 37°47′ and 43°02′ South (Table S1, Fig. 1). Specimens were collected under authorizations SAG-1898 and SAG-4729 from Servicio Agrícola y Ganadero (SAG). All methods involving *L. pictus* were carried out in accordance with a protocol reviewed and approved by the Ethics Committee of the Fondo Nacional de Ciencia y Tecnología (FONDECYT, Chile) and the Ethics Committee of the Universidad de Concepción (UDEC, Chile), as part of the review process for the Fondecyt Research Grant 1090664. Voucher specimens were catalogued in the Museo de Zoología at Universidad de Concepción (MZUC (UCCC), Chile). No specimen was found in the Chilean “Longitudinal Valley”, very likely due to the almost complete replacement of *Nothofagus* forests by non-native *Pinus* dominated agro-forestry systems, which has probably eradicated *L. pictus* populations from this region [23]. As such, our sampling consists mainly of localities in both the Andean and the Coastal mountain ranges and includes the distributional range of four of the six currently recognized subspecies [37]. We lack samples of *L. pictus codoceae* and *L. pictus talcanensis*, whose status has been questioned [24].

### DNA sequence acquisition

Of the 196 mitochondrial sequences analyzed, 59 were retrieved from Genbank (EU649356–EU649413, AY367791) and the other 137 were generated in this study. Total genomic DNA was extracted from muscle tissue preserved in ethanol using the Wizard SV Genomic DNA Purification kit (Promega). A fragment of the mitochondrial cytochrome b (cyt-b) gene was amplified using primers GluDGL (5′-TGACTTGAARAACCAYCGTTG-3′), F1 (5′-TGAGGACARATATCHTTYTGRGG-3′) y cytb3 (5′-GGCAAATAGGAARTATCATTC-3′). PCR amplifications of cyt-b were carried out as described in Victoriano et al. [28]. The nuclear genes EXPH5 and LDB5B were amplified following previously published procedures [38], [39]. Negative controls were used for all amplifications. Amplicons were purified using the GenClean III kit (BIO 101, Inc) and later sequenced at Macrogen Inc. (Korea). Sequences were edited with Codon Code Aligner 2.0 [40], and deposited in Genbank under accession numbers JX494826-JX494964 (cyt-b) and JX999516-JX999585 (nuclear genes). Nuclear LDB5B and EXPH5 alleles at heterozygous sites were inferred with PHASE 2.1 [41], [42], [43]. We conducted ten replicate PHASE runs using 1000 burn-in steps and 1000 iterations. The PHASE probability threshold was set to 0.60, a level that has been suggested as optimal for reducing the number of unresolved haplotypes with fewer false positives [44]. To test for recombination, we used multiple tests implemented in RDP3 [45].

### Genealogical analyses

Sequence alignments were performed with Clustal X [46] using the default values for all alignment parameters. Mitochondrial DNA sequences were translated to check for sequence functionality. For both nuclear and mitochondrial genes, the degree of saturation of the matrix was assessed following the procedure described in DAMBE 5.0.11 [47]. The test was done with 100 replicates using the proportion of invariant sites found by jModeltest 0.1.1 [48].

The cyt-b gene tree was constructed with Bayes Phylogenies 1.1 [49], based on the best-fit model of sequence evolution estimated with jModeltest 0.1.1 and the BIC criteria. The phylogenetic tree was rooted, using as outgroup the combined haplotypes of *Liolaemus chilensis* and *Liolaemus cyanogaster*, both closely related to *L. pictus* [22]. Bayesian searches were run for 10×10^6^ generations and sampled every 500 generations; the first 2000 trees were discarded as burn-in, and a majority rule consensus tree of the remaining sampled trees was constructed with Bayes Trees 1.0 [50]. Relationships among haplotypes were also explored with statistical parsimony (SP), using TCS [51], with the inferred network parsimony confidence limit of 95%. In order to polarize haplotype states for the nuclear gene networks, we included alleles of the closely related species of the same genus [22] *L. lemniscatus* and *L. chiliensis* for LDB5B, and of *L. lemniscatus* and *L. tenuis* for EXPH5. From the haplotype network we inferred the ancestral haplotype according to the criteria of Templeton et al. [52]. Coalescent theory predicts that the haplotypes from the periphery of a network are more recent in origin than the central haplotypes, and we followed this prediction in our interpretations of the nuclear haplotype networks in relation to the outgroup alleles. We then used the ancestral-derived network position of the alleles within their geographical context to test the prediction that refugial areas should include a relatively high frequency of ancestral haplotypes, following the methods of Miraldo et al. [36].

### Genetic structure and demographic analyses

For the mtDNA dataset, population diversity estimates, including nucleotide diversity (*π*), number of haplotypes (h), haplotype diversity (H_d_) and number of segregating sites (s), were calculated using DnaSP 4.5 [53]. These indexes were not estimated for the nuclear genes. Observed pairwise genetic distances (uncorrected p-distance) for cyt-b sequences were estimated for all samples and within and between clades using MEGA 5 [54]. An analysis of molecular variance (AMOVA) was performed using Arlequin 3.11 [55] by grouping the haplotypes by localities and main clades. In addition, the neutrality test of Fu [56] was applied to these clades as a way to assess their recent demographic history. These tests were performed with DnaSP 4.5. Analyses of mismatch distributions of pairwise haplotype comparisons and estimations of the raggedness index (Rgg) were achieved with Arlequin 3.11. The raggedness index takes high values (Rgg>0.05) when pairwise distributions are multimodal, which is expected for samples gathered from stable populations, and low values (Rgg<0.005) for unimodal distributions, a signal of recent population expansion [57]. In addition, we estimated the probability (p Ragg) of observing a higher estimated raggedness value than the observed value under the hypothesis of population expansion, as implemented in Arlequin 3.11.

For cyt-b, the spatial structure of the genetic variation was assessed with a Bayesian approach using Geneland [58]; the goal was to identify subgroups within the main clades, which later were considered as separate demographic units for the analysis of migration between populations. Geneland provides a Bayesian probabilistic frequency distribution graph that indicates the most likely number of populations and their geographic limits. We ran a non-correlated spatial model for haploid information with three independent chains run for 15×10^6^ generations, sampling every 250 generations. For complementarity purposes, spatial patterns of genetic divergence between sampling areas across the range of *L. pictus* were analyzed using two additional procedures. For the mtDNA gene, we performed a genetic landscape shape interpolation procedure using the program Alleles In Space [AIS; 59]. This method, based on Delaunay triangulations, produces a three-dimensional graphical representation of genetic distance patterns across the full landscape analyzed in a study. This approach allows a better visualization of the heterogeneity of the genetic divergence patterns across a landscape, and has proven useful in phylogeographic studies [60]. Genetic landscape shape interpolation analyses were carried out using both “raw” genetic distances and residual genetic distances as described in the AIS software information. The residual genetic distances are derived from the linear regression of all pairwise genetic distances on geographical distances, allowing removal of the effect of geographical distance. Isolation-by-distance (IBD) was also tested following the procedure implemented in AIS.

Historical changes in Ne for each of the main clades were evaluated using graphic reconstructions of population sizes through time estimated with a lineage-through-time (LTT, Bayesian skyline plot analysis) plot using BEAST 1.4.7 [61], under a relaxed molecular clock. Runs consisted of 15×10^6^ generations with sampling every 1000, and the first 10% discarded as a burn-in. Because the cyt-b rate of evolution for *L. pictus* was unknown, it was estimated with BEAST 1.4.7 using a relaxed molecular clock in a phylogenetic framework. In order to estimate the age of the main nodes on the cyt-b tree and analyze the association between the geographic distributions of the haplogroups and their ages, we conducted an analysis with a Yule process of speciation using the software BEAST 1.4.7 [61]. To place the mean priors in the tree we used two fossils from the subgenus *Eulaemus*, both from Argentina. One fossil correspond to 18.5–20 Mya [62] while the second is a recent fossil associated with the origin of *Liolaemus multimaculatus* 70,000 ya [63], [64]. The model used for this analysis was the TN93 with a relaxed uncorrelated log normal clock selected with a likelihood value of −3981.282 using Bayes factor in Tracer 1.5 [65]. The length of the chain was of 40×10^6^ steps, sampling parameters every 1000 steps and discarding the first 10% of them. We obtained a substitution rate of 6.57%/million years, which was the same used for the Bayesian skyline plot analysis.

The magnitude and spatial arrangement of gene flow was assessed with the coalescence approach implemented in Migrate 3.27 [66], based on a Maximum Likelihood algorithm. These analyses were based on the population clusters defined by Geneland. Within each of the two main clades (see below) we estimated directional gene flow between clusters and, when a cluster included widely distributed localities, we further delimited geographic areas in order to estimate directional gene flow between physiographic regions (e.g. Coastal mainland vs. high-Andes). We used the following base frequencies A = 0.3010, C = 0.2742, G = 0.1361, T = 0.2887, and Ti/Tv = 15.1109, and performed analyses with 10 short chains with sampling increasing to 200 and 500 recorded genealogies, and one long chain with sampling increment of 100 and 40,000 recorded genealogies. For all runs the first 20,000 genealogies were discarded as burn-in. The criterion of Gelman was used to verify that sampling was made from the stationary distribution.

### Ecological-niche modelling

We conducted an ecological niche modelling analysis in order to identify potential refugia for *L. pictus* during the LGM and determine if these predicted areas of past occurrence were concordant with those inferred in the phylogeographic inferences. Ecological niches and potential geographic distributions were modeled using the maximum entropy method [67]. Climatic envelopes were estimated from 19 environmental variables that likely summarize niche dimensions that are particularly relevant to determining species distributions [68]. For the LGM and present climate conditions, we used two sets of monthly climate data (precipitation and temperature). For current conditions (means 1950–2000), we used WorldClim, a global climate database with a spatial resolution of 2.5 min [69], available at http://www.worldclim.org. Maxent generates Ecological Niche Models (ENM) using presence-only records, contrasting them with pseudo-absence data sampled from the remainder of the study area. The present-day ENM were developed based on the 123 confirmed occurrence points for *L. pictus* [23]. The bioclimatic niche of the species was then projected on to past climate layers to predict the potential range of the species during the LGM (∼21,000 ya). We used the default convergence threshold (10^−5^) and maximum number of iterations (500), using 25% of the localities for model testing. As suggested by Waltari et al. [68], we chose a presence threshold to render each projection into a binary form, and considered grid cells with a cumulative probability of more than 10 (from a range of 0–100) as suitable. This threshold identified smaller areas than a lowest-presence threshold that yielded zero omission error, thus resulting in a more restricted picture of potential distribution.

The inputs of all the analyses were deposited at Dryad (http://dx.doi.org/10.5061/dryad.853t3)

## Results

### Genealogical analyses

We obtained an alignment of 644 base pairs of the cyt-b gene from 196 sequences gathered from specimens of *L. pictus* collected at 46 localities. Results of the test of Xia & Xie [45], including the outgroups, indicated a low degree of saturation. We found a total of 130 haplotypes and 194 segregating sites. The genetic and nucleotide diversity were Hd = 0.9907±0.0023 and *π* = 0.0575±0.0018, respectively (Table 1). Pairwise genetic distance between localities ranged from 0.000±0.000 to 0.1140±0.013 (average 0.0575±0.0049). The zero values correspond to comparisons between localities from the southernmost mainland range close to the Andes (i.e. 28–29, 28–30, 29–30), while the highest value corresponds to the comparison between localities 8 (Salto El Indio, Malalcahuello, in the northern distribution close to the Andes at 38°25′S) and 16 (MN Alerce Costero, Coastal range at 40°10′S). A list of the haplotypes per sampling site is given in Table S2. Of the 130 unique haplotypes found, only 13 were shared among localities. Twenty of 46 localities included at least one shared haplotype. Twelve localities presented a single shared haplotype and 2 sites shared 3 haplotypes. Most of the shared haplotypes were detected in the central and southern range of the distribution of the species.

**Table 1:** Estimates of haplotype diversity (Hd), nucleotide diversity (*π*), Fu's Fu test with associated levels of significance (*: <0.05, **: >0.05), and raggedness index (Rgg) for the two main phylogroups described by the Bayesian phylogeny.

Main clades	N	s	h	Hd	*π*	Fs	Rgg
Northern Clade	44	103/644	30	0.986	0.03243	−3.442*	0.0013 **
Southern Clade	152	161/644	100	0.987	0.04042	−3,987*	0.0019 **
Total	196	194/644	130	0.991	0.05750	ne	ne

N: sample size, s: number of segregating sites, h: haplotype number, ne: non estimated.

**Figure 2:**
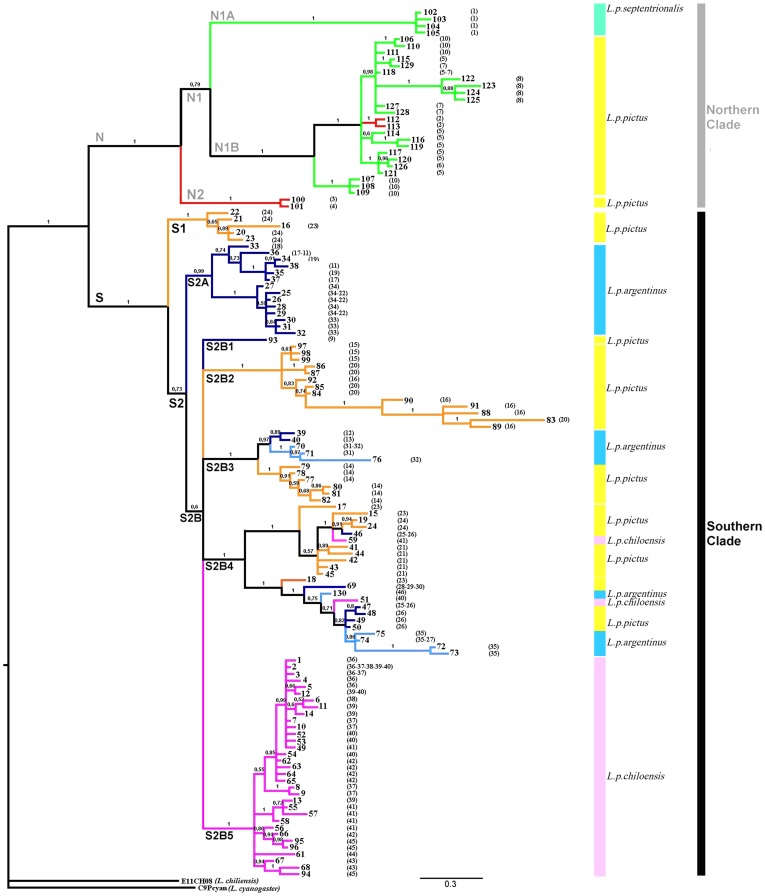
Cyt-b genealogy obtained in a Bayesian analysis. Branch colors as codes in the map of Fig. 1. Numbers on tips of branches correspond to haplotypes detailed in Table S2. Numbers in parentheses are localities, grey and black bars identify the two main clades, and colored bars represent the subspecies of *L. pictus*.

Phylogenetic reconstruction of the unique cyt-b sequences recovered two well-supported reciprocally monophyletic clades of *L. pictus* haplotypes corresponding to northern and southern populations (Fig. 2). The northern phylogroup (clade N) was composed of haplotypes from samples collected between 36°46′S and 38°26′S in the Coastal Cordillera and the Andean Cordillera. This clade was subdivided into two well-supported subclades: one (N1) includes haplotypes from both the Andes and the Coastal Cordillera and the second (N2) including haplotypes from two localities near Purén in the Coastal Cordillera. Within the former, haplotypes from subclade N1A were all from the region of Las Trancas in the northern Andean Cordillera, which corresponds to the distributional range of the subspecies *L. pictus septentrionalis*. Subclade N1B was widely distributed, weakly resolved, and included haplotypes from the Coastal Cordillera and the Andean Cordillera (Fig. 2).

The southern phylogroup (Clade S) was composed of two strongly differentiated groups (S1 and S2), the smallest of which (S1) was strongly supported statistically and included only haplotypes from a restricted area from the Chilean Coastal range located at 41°30′S. The larger clade (S2) was composed of two subclades (S2A and S2B) having a moderate probabilistic support. Subclade S2A was well supported and included haplotypes from the Western Andes (e.g. Antillanca and Puyehue) from 39°S to 41°S. These localities are within the Chilean distributional range of the subspecies *L. pictus argentinus*. The larger subclade (S2B) was a weakly supported polytomy of five subclades. The smallest of these, S2B1, was composed of one haplotype shared by individuals from the Andean locality of Malalcahuello (Western Andes), an area geographically very close to some localities harboring haplotypes from the Northern clade. Subclade S2B2 included five haplotypes with the longest branch lengths of the whole sample, most of them from a small region of the Coastal range corresponding to localities 16 (MN Alerce Costero) and 20 (Close to Heicolla). The two subclades S2B3 and S2B4 were composed of haplotypes broadly distributed between western Argentina, the southern Chilean Andes and the Coastal range. Finally, subclade S2B5 was well supported and composed exclusively of haplotypes from Chiloé Island. Only two haplotypes collected at localities 40 and 41 in the north of the island were not in this clade; both were part of clade S2B4. Thus, subclades S2B4 and S2B5 overlap on northern Chiloé Island.

The AMOVA showed that 55.5% of the total cyt-b variation was explained by differences between the two main clades (Table S3). The number of segregating sites and the haplotype richness were lower in the Northern clade than in the Southern clade (103 vs 161 and 30 vs 100, respectively; Table 1), and the respective values of haplotype richness standardized for sample sizes were 0.68 and 0.66. Haplotype diversity values (Hd) were very similar between both clades, while nucleotide diversity was higher in the Southern clade (Clade S π = 0.04042, Clade N π = 0.03243). The average genetic distance between the Northern and Southern clades was 0.090±0.009.

In general, all pairwise p-distance values involving comparisons between Northern and Southern clades are higher than values from comparisons among the Southern subclades. In addition, the among-Northern subclade values are higher than among-Southern subclade values (Table S4).

Mean ages and 95% highest posterior density of mtDNA phylogroups are shown in Table 2. Basal divergence within *L. pictus* (N+S) is estimated to have started approximately 751,000 ya (547,000–988,000) in the mid Pleistocene. Clade N is estimated to have diverged approximately 523,000 ya (338,000–720,000), and clade S about 580,000 ya (424,000–753,000), during the second half of Pleistocene (Table 2).

**Table 2:** Divergence time estimates in million years (Ma) from the most recent common ancestor (mrca) of all phylogroups of *L. pictus* recovered in the phylogenetic analysis using Beast. Codes of mtDNA phylogroups as in tree in Fig. 2.

mtDNA phylogroups	Lower HPD	Mean	Upper HPD
All (N+S)	0.547	0.751	0.988
N	0.338	0.523	0.720
N1	0.284	0.440	0.614
N1A	0.015	0.072	0.147
N1B	0.181	0.288	0.411
N2	0.002	0.055	0.143
S	0.424	0.580	0.753
S1	0.065	0.187	0.342
S2	0.383	0.524	0.671
S2A	0.161	0.309	0.475
S2B	0.352	0.475	0.608
S2B2	0.198	0.305	0.419
S2B3	0.125	0.235	0.351
S2B4	0.226	0.333	0.446
S2B5	0.152	0.251	0.363

**Figure 3:**
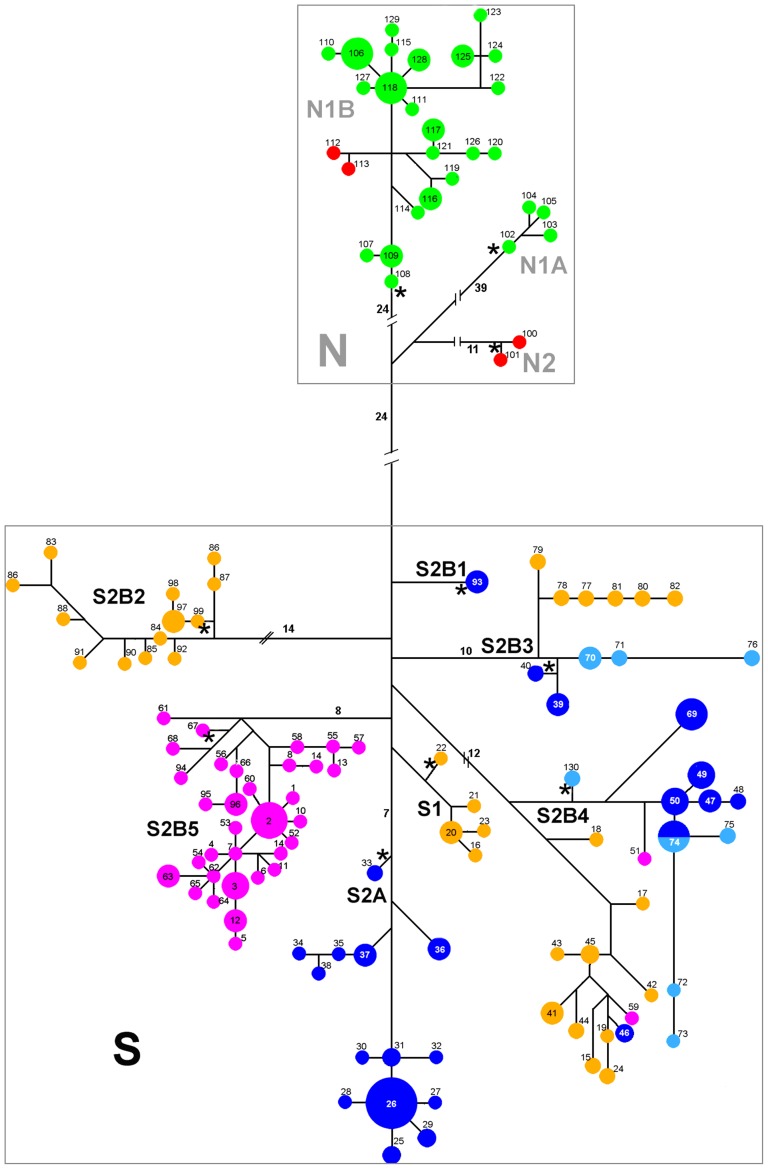
Cyt-b haplotype network for *L. pictus*. Colors are the same as in Fig. 1, probable ancestral haplotypes for each haplogroup are labeled with asterisks, and numbers on the lines correspond to mutation steps.

The cyt-b haplotype networks are highly congruent with the Bayesian genealogy. The Northern and Southern haploclades are recovered as separate networks (Fig. 3), and the Northern haplogroup includes haplotypes from Coastal and Andean regions that are closely linked except for two subgroups of haplotypes that appeared well differentiated. One of these includes samples from the Andean region of Las Trancas and the second represents the coastal region of Purén. The Southern haplogroup includes samples from the Coastal Cordillera, the Andean Cordillera, and Chiloé Island. Haplotypes from the Coastal and Andean Cordilleras are mixed in this network, and while most haplotypes from Chiloé Island form an exclusive group, two insular haplotypes are associated with haplotypes from the mainland. The root of the cyt-b network should be between the highly divergent N and S clades, and this allowed us to infer the ancestral haplotypes within each haplogroup (Fig. 3). The inferred ancestral haplotypes from the mtDNA network showed the shortest branch lengths and the smallest number of changes from the root in the Bayes tree (Fig. 2).

Overall, from the 10 clades revealed in the cyt-b network, four suggested ancestral haplotypes were from the Coastal range, five from localities in the western Chilean Andes, and one from the Eastern Argentinean Andes. Among the six Andean ancestral haplotypes, two were distributed in areas outside of the LGM ice shield boundaries, whereas the other four fell within this border. For detailed distribution of ancestral haplotypes see Table S2.

**Figure 4:**
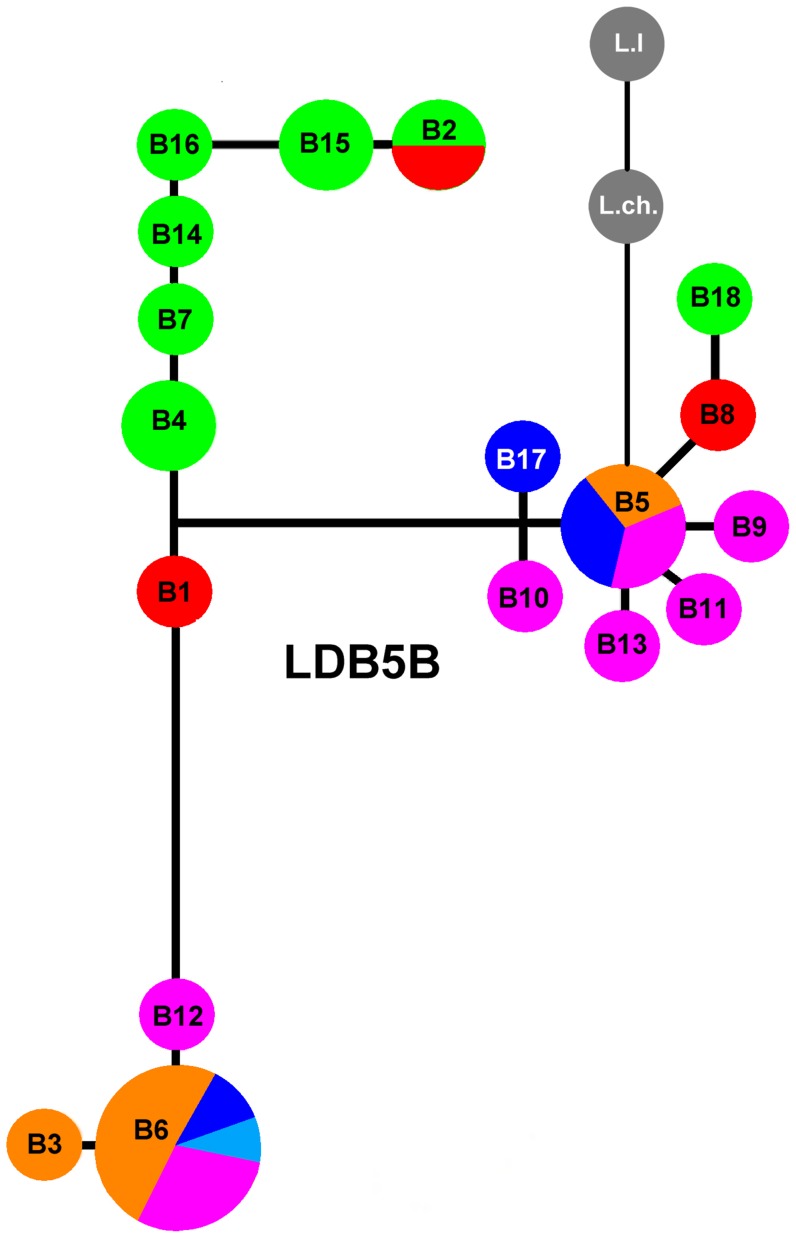
Haplotype networks for the nuclear gene LDB5B in *L. pictus*. Grey haplotypes correspond to the outgroups *L. lemniscatus* (L.l) and *L. chiliensis* (L.ch). Color codes as in the map of Fig. 1.

The nuclear gene networks (Fig. 4 and Fig. S1) were partially congruent with the cyt-b network; 18 haplotypes were detected for each gene. The LDB5B network (Fig. 4), was most congruent with the mtDNA network and resolved three groups: one corresponded exactly to the Northern clade of mtDNA haplotypes, and two divergent clades were composed of haplotypes from localities that were part of the Southern mtDNA clade. Although the LDB5B alleles did not form reciprocally monophyletic geographic groups, contrary to what observed on the cyt-b gene tree, there was a clear tendency for the Northern haplotypes to form a single group. The position of the outgroups in this network suggests that haplotype B5, broadly distributed, is ancestral. The EXPH5 network did not recover discrete clades, rather all haplotypes are organized in a linear array of similar distances with Northern haplotypes at one end (Fig. S1). The position of the outgroups in this network suggests that haplotypes E5 and E6 from the Western Andes are ancestral. In general terms, the samples of the Northern mtDNA clade (clade N) tend to also form a unique cluster in both nuclear gene networks, although they were not exclusive. The haplotypes in both nuclear genes showed much less structure relative to the mitochondrial network.

### Demographic analysis

Analysis of the population genetic structure using the program Geneland revealed the presence of four populations within the mtDNA Clade N (named Northern clusters A–D in the Migrate analyses) and three within Clade S (named Southern clusters A–C in the Migrate analyses). The general distributions of these clusters are detailed in the Table 3.

**Table 3:** Results of Θ and migration rate between clusters of population of *L. pictus*. Clusters correspond to the same already defined by Geneland.

				Gene flow	(Nm)	
Northern Clade clusters _r_	N	θ	A	B	C	D
Coastal-Andean _r_ (A)	22	0.0235	-	2.103	1.81E-09	1.58E-11
Andean _r_ (B)	5	0.0069	6.46E-13	-	6.46E-13	6.46E-13
Coastal-Andean _r_ (C)	13	0.0124	1.092	7.51E-10	-	1.67E-10
Andean _r_ (D)	4	0.0044	0.119	1.78E-12	1.78E-12	-
**Cluster North A**	**N**	**θ**	**a**	**b**		
Coastal mainland _r_ (a)	2	0.0026	-	0.4915		
Andean _r_ (b)	20	0.0136	3.02E-03	-		
**Cluster North C**	**N**	**θ**	**a**	**b**		
Coastal mainland _r_ (a)	2	0.0030	-	0.0996		
Andean _r_ (b)	10	0.0093	3.26E-07	-		
**Southern Clade clusters _r_**	**N**	**θ**	**A**	**B**	**C**	
Chiloé- Coastal mainland-Andean _r_ (A)	120	0.1374	-	1.403	1.167	
Coastal mainland-Andean _r_ (B)	22	0.0560	4.73E-09	-	6.313	
Coastal mainland _r_ (C)	11	0.0350	1.807	1.10E-07	-	
**Cluster South A (comparison 1)**	**N**	**θ**	**a**	**b**		
Coastal mainland _r_ (a)	23	0.0665	-	2.99E-03		
Andean _r_ (b)	46	0.0215	16.754	-		
**Cluster South A (comparison 2)**	**N**	θ	**a**	**b**		
Chiloé Island _r_ (a)	50	0.0408	-	11.636		
Coastal mainland-Andean _r_ (b)	69	0.0706	3.45E-09	-		
**Cluster South A (comparison 3)**	**N**	**θ**	**a**	**b**		
Chiloé Island _r_ (a)	50	0.0480	-	2.29E-03		
Andean _r_ (b)	46	0.0223	15.881	-		
**Cluster South B**	**N**	**θ**	**a**	**b**	**c**	
West Andes _r_ (a)	44	0.0116	-	0.9011	0.9593	
East Andes _r_ (b)	10	0.0140	7.17E-13	-	2.72E-12	
Coastal mainland _r_ (c)	25	0.0260	1.00E-12	1.1936	-	

Θ = 2Neµ. N = number of individuals in each sample. Migration rate values are in Nm. _r_ = receiving population.

**Figure 5:**
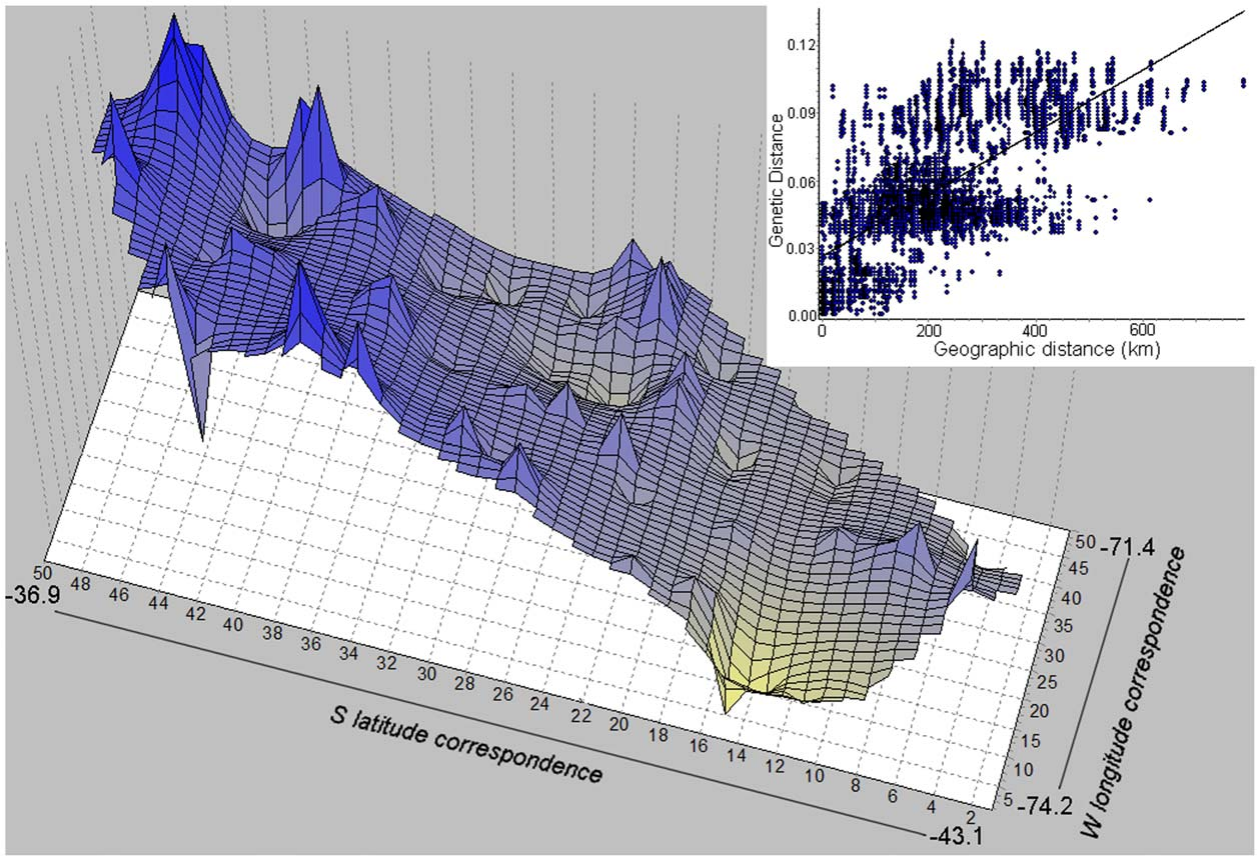
Genetic landscape shape (GLS) interpolation analysis and Isolation by Distance analysis, for *L. pictus*. GLS was made by using a 50×50 grid and a raw genetic distance. *X* and *Y* axes correspond to geographic locations within the overall physical landscape examined in this study (Fig. 1). Surface plot heights reflect genetic distances.

**Figure 6:**
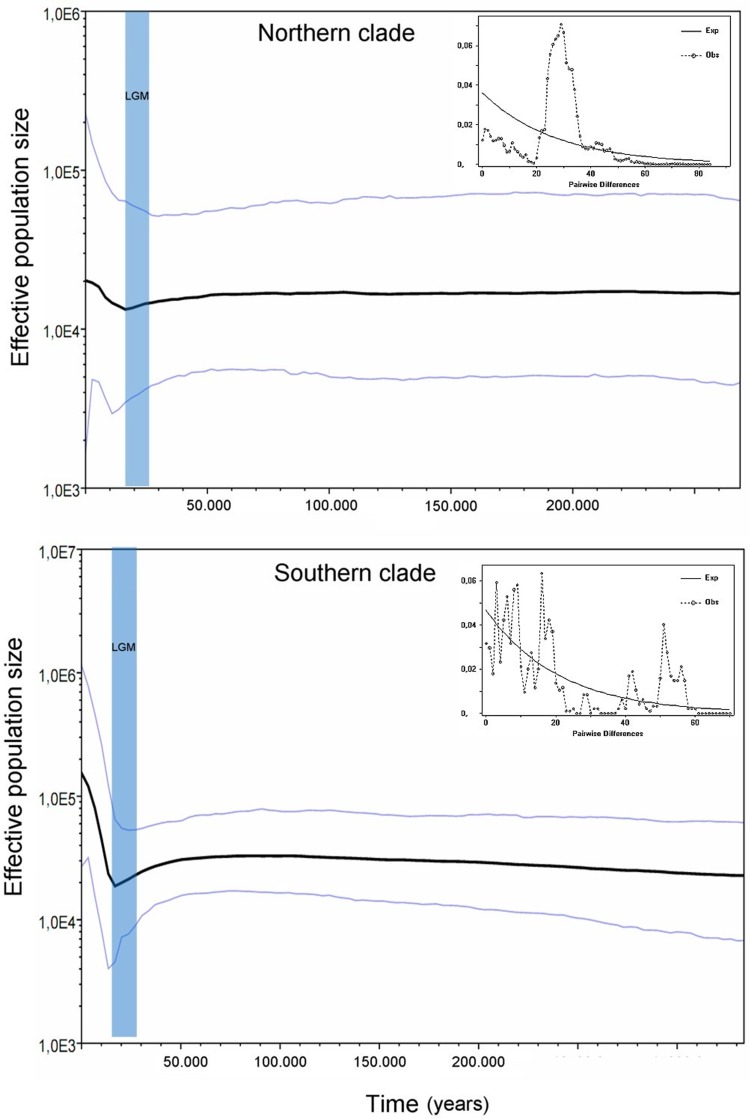
Prediction of the historical effective population size in *L. pictus*. Bayesian skyline plots and mismatch analysis for the two major mtDNA clades of *L. pictus*. The black line is the temporal trend of the mean of effective population size. Vertical light blue band corresponds to the LGM.

The analysis of isolation-by-distance revealed a high and highly significant positive correlation between geographic and genetic distances (*r* = 0.716, *p*<0.001). Despite the strong correlation for the total pairwise comparisons, three clouds of spots could be detected (Fig. 5). One of these clouds shows high genetic distances both for geographically close and distant comparisons, and included the between N-S comparisons. This pattern was confirmed by the genetic landscape shape interpolation analyses for which no strong discontinuity could be observed from South to North (Fig. 5), although high atypical peaks were associated with the Northern clade (N). This graphical representation shows more genetic homogeneity among southern localities than among those of the northern part of the range. Demographic analyses showed signals of recent population expansions as expected for the Southern group but also for the Northern group, and Fu's neutrality tests gave significant negative Fs values for both (Table 1). Mismatch distribution analyses provided values of the raggedness index for each group below 0.05 and with *p* values higher than 0.05 (Table 1), indicating non-significant differences between the expected unimodal distribution and the observed distribution of pair-wise differences between haplotypes. Bayesian skyline plot analyses showed a reduction of N_e_ in both the Northern and the Southern group; although as expected, oscillations are more pronounced for the latter (Fig. 6). The N_e_ reduction in both clades started about 50,000–60,000 ya, and the lowest N_e_ values were estimated around 16,000–17,000 ya. The Southern group showed a greater magnitude in both reduction and recovery of population size, although both groups show rapid and pronounced recoveries of N_e_ with no signal of a slow down in this trend in the Southern group.

The assessment of gene flow among the groups within each clade revealed a mixed pattern (Table 3). In the clade N, there is greater evidence of historical gene flow from Andean to Coastal populations. For the Southern clade, the highest level of gene flow is estimated from cluster C towards cluster B (Nm = 6.313), with both clusters composed of populations from Coastal mainland and Andean localities. Globally, for all 15 comparisons the estimates of gene flow could be considered as non-zero values. Among these comparisons, five revealed predominant gene flow from Andean localities lying within the LGM ice shield boundaries towards Coastal localities, but as predicted we find a clearly higher value of gene flow from Coastal to Andean populations only for the Southern cluster A.

### Ecological niche modelling

**Figure 7:**
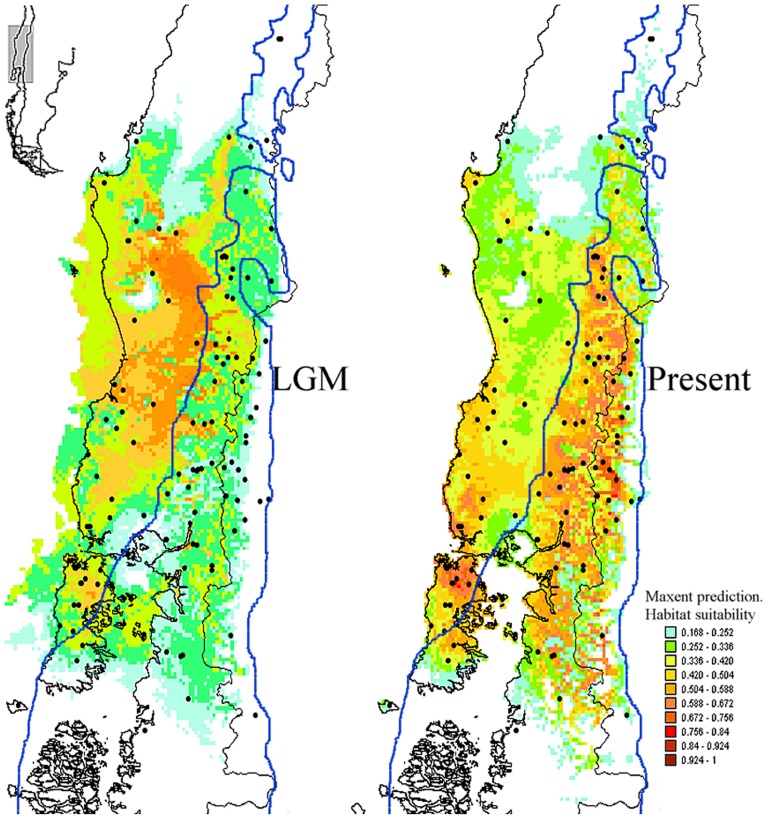
Present and LGM potential distribution for *L. pictus* estimated from Ecological Niche Modeling (ENM). The color scale corresponds to the probability for suitable habitat; red and light blue indicate highest and lowest suitability values respectively. The heavy blue line delimits the maximum extension of ice shield during LGM according to Heusser [11].

Ecological niche modeling predicts several fragmented refugia for *L. pictus*, including at both lowlands and Andean valleys. The Maxent model predicts a current distribution close to the one known for *L. pictus*, from the Andes of the Maule Region in Central Chile (ca. 36°S) to the Chiloé Archipelago and nearby mainland in Southern Chile, as well as the temperate forest of the Argentinean Rio Negro and Chubut Provinces (Fig. 7). The habitat suitability landscape shows current high habitat probabilities for *L. pictus* in the Andean range throughout most of the latitudinal distribution, and in Coastal areas in southern Chile and Chiloé Island. The LGM distribution predicted for *L. pictus* was fragmented and not totally excluded from the Andes. The southern half of Chiloé Island, the mainland to the east of this island and the highest elevations in the Southern Andes were generally not suitable for *L. pictus*. During the LGM increased habitat suitability is predicted for the lowlands in the central distribution of *L. pictus* in the Longitudinal Valley, and a refugium is predicted for the north end of Chiloé Island. Some small east-west areas following valleys in the west flank of the Andean Cordillera, and within the presumed boundaries of the large ice shield, may also have remained suitable. According to the predicted sea level during the LGM, the potential distribution of *L. pictus* could have been extended west of the present coastline throughout most of the species latitudinal range. The central and northern parts of the current distribution of *L. pictus* fit well with the past potential distribution, except where conditions were fragmented in the Andes during the LGM.

## Discussion

The analysis of the mitochondrial and nuclear genetic variation of *Liolaemus pictus* across its distribution revealed a complex phylogeographic pattern and demographic history. Results indicate a mix of divergence due to isolated refugia followed by dispersal from them. Our results show the effect of recent glaciations on *L. pictus*, however the predicted effect was not unambiguous for southern and Andean populations. In fact, evidence of dispersal both from glaciated and non glaciated source areas was found, along ancestral haplotypes distributed both in the Andean and Coastal ranges.

### Phylogeography

Our analyses show a complex phylogeographic history, considerable genetic structure, and signals of complex patterns of gene flow in *L. pictus*. Our mtDNA gene tree recovers two large and deeply divergent groups: a Northern group (clade N) distributed from the northern extreme of the species range at ∼37°S to 39°S, and a Southern group (clade S) with a broader distribution that includes most of Chiloé Island. Both clades include Coastal and Andean localities and overlap in a small area close to Malalcahuello in the western Andean piedmont (localities 8 and 9 in Fig. 1). The northern group is heterogeneous, with well-differentiated and deeply divergent internal subgroups, whereas the southern group is more homogeneous (see p distances in Table S4). In general, genetic diversity was higher at southern localities, and spatial analyses showed that this group, despite having higher haplotypic diversity, had a weaker geographic structure than the northern clade. For example, it is not possible to clearly differentiate internal areas, even when considering large and geographically distant regions such as East and West of the Andes, the Coastal Cordillera, or Chiloé Island (see also [28]). These analyses, along with the IBD analyses, suggest a rather continuous distribution of the genetic divergence throughout the range of *L. pictus* southern clade. However, these results must be interpreted with caution because they may be reflecting the differences between the highly differentiated clades rather than a specific pattern within these clades, as indicated by the three groups in the IBD scatterplot.

The deepest splits between subclades were observed in Clade N; the MRCA of the phylogroup N1A from the northernmost locality in Las Trancas was 72,000 ya (15,000–147,000 ya), but the split from its sister clade N1B was 440,000 ya (284,000–614,000 ya). Similarly, clade N2 from the coastal range in Purén diverged 55,000 ya (2,000–143,000 ya), but its split from sister clade N1 was 523,000 yr (338,000–720,000 ya). These divergence levels suggest strong fragmentation, low connectivity and reductions of Ne, especially in clades N1A and N2. Although the effects of the glacial cycles were less prominent in the North, *Nothofagus* forests are more fragmented and exposed to changes in size because those areas are close to the limits of their distribution [4], [23]. Thus, while the effects of the glaciers in these regions may have been minimal, the marginal conditions of the habitat may have promoted fragmentation, differentiation, and unstable population sizes in *L. pictus*. In contrast, genetic distances within clade S are lower and the clade is characterized by shorter branch lengths, suggesting a greater level of dispersal throughout this area. Although the genetic distances between haplotypes from Chiloé Island and the surrounding mainland populations are low compared to those from the Northern clade, almost all haplotypes from this island constitute a well-supported monophyletic group. Only two cyt-b haplotypes (59 and 51), from Northern Chiloé, are recovered in an otherwise mainland phylogroup within subclade S2B4. Within S2B4, other closely related haplotypes are from Argentina (Aluminé and Bariloche), and from both Coastal and Andean Chilean regions in southern Chile. Some of these haplotypes are shared by more than one locality and with a broad distribution (e.g. 46, 47, 69 and 74), which suggests an historical connection between populations from the east and west of the Andes and Chiloé Island. Our results disagree with Vidal et al. [24], because they do not infer immigration from the mainland to Chiloé Island, rather several of our Nm values suggest bidirectional gene flow (Table 3). The age of the mainland clades, which include haplotypes from Chiloé Island, implies that gene flow to and from the island must have occurred prior to the LGM. Clade S2B4, which includes the two Chiloé Island haplotypes, is 333,000 yr old, and the divergence times for these two haplotypes are approximately 80,000 to 100,000 ya (estimation not shown). This suggests that immigration from the continent occurred prior to the LGM, in response to the formation of land bridges linking the island with the mainland during previous glacial periods. However, the extent of migration is not consistent with the scenario of falling sea level during the repeated glacial cycles that have connected the northern part of the island of Chiloé during the Quaternary [70], and which have resulted in a land bridge between the northern end of the island and the continent (Chacao Channel). In fact, several authors have proposed the existence of recurrent bridges between Chiloé Island and the continent associated with a drop in global sea level during glacial periods [71], [72], [73], [74], [75]. Although such recurrent events should facilitate the migration of organisms, *L. pictus* dispersal could have been limited by the extensive ice cover during the LGM, especially on the east side of the island. Retreat of this ice shield may have also produced abundant meltwater, and such geologically recent fluvioglacial barriers may have prevented island-mainland gene flow. Therefore, this process would have contributed to reduce the connectivity between Chiloé Island and the continent for *L. pictus* rather than to increase it. This highlights the importance of considering the temporal dynamics throughout each glacial cycle if one wants to understand the effects of the glaciations on gene flow.

In addition, connectivity between Chiloé Island and the continent could have been limited because the exposed continental shelf may not have been colonized by a continuous *Nothofagus* forest, due to different physical and chemical properties of the soils which were impacted by marine sediments for a long time. Although *L. pictus* inhabits the borders of *Nothofagus* forests, it needs high density, high connectivity, and large size patches of arboreal vegetation [20], [76]. Nuñez et al. [14] studied the frog *Eupsophus calcaratus*, a species also associated to the temperate *Nothofagus* forest, and recovered a monophyletic group for the haplotypes from Chiloé Island which was the sister clade of samples from Alerce Andino in the continent (our localities 25 and 26), suggesting historical dispersal between both areas. For larger vertebrates such as the southern deer *Pudu pudu*, there is evidence for high divergence, low gene flow and reciprocal monophyly between Chiloé Island and the mainland [77]. The split for both deer clades was estimated to approximately 437,000 ya, which is similar to our estimate for *L. pictus* (divergence time from MRCA of clade S2B, 475,000 ya). Independent of the presence of small or medium sized fluvial barriers during glacial periods, this species should have been able to move between the island and the continent if the forest had colonized continuous formations over the exposed shelf, thereby erasing the pattern of reciprocal monophyly detected in our study. For Chiloé Island, we found no evidence for recent or post-LGM migration from the northwestern non-glaciated area to the south of the “Chiloé ice diagonal” (Fig. 1), suggesting that vagility for *L. pictus* may have been low. Similarly, there is no evidence of shared haplotypes between these two areas in the frog *Eupsophus calcaratus* [14].

For samples from clade S, recent gene flow is inferred from the occurrence of shared haplotypes among several localities, all of which include Southern clade and Andean sites within the maximum limits of the LGM ice sheet. Some haplotypes are shared among nearby localities, including sites 36–40 from northwestern Chiloé Island. Antillanca (site 34) and Puyehue (site 22) share three haplotypes in the Western Andes. However, we found evidence for recent gene flow between localities more than 100 km apart, such as between Río Puelo and Bariloche (sites 27 and 35 respectively), Huerquehue and Los Llolles (11 and 17), and Chaitén and Canal Garrado (28–30). The last example suggests a more recent connection between mainland localities surrounding Chiloé Island than between this island and the continent. Prior evidence for post-LGM dispersal in the glaciated Andes has been found in other species like the freshwater crab *Aegla alacalufi* [13], the freshwater fish *Galaxias maculatus* [78], and the tree *Araucaria araucana* [79]. For *G. maculatus* and *A. araucana*, re-colonization occurred from Eastern Andean refugia [78], [79].

Despite the exhaustive sampling effort over the entire range of *L. pictus* [23], no specimen was found in the Longitudinal Valley. The complete loss of native forest, especially of trees of the genus *Nothofagus* [80], has eliminated suitable habitat for *L. pictus* in the BioBío and Araucanía regions and almost certainly led to the extinction of *L. pictus* populations in these regions [81], [82]. Given the substitution rate of the mitochondrial gene employed in our study, the time passed since these extinctions was probably insufficient to detect a significant effect on population structure [83].

Co-distribution of haplotypes from highly divergent clades suggests older migration between different areas. For example, sites 23 and 24 from the Estaquillas area (close to the mainland coast in southern Chile) included S1 haplotypes which are endemic to that area, and haplotypes from the broadly distributed clade S2B4. Similarly, sites 40 and 41 along the northern margin of Chiloé Island included haplotypes from the same clade S2B4 and from clade S2B5, which includes only haplotypes endemic to the island. If the secondary contact hypothesis is valid, and excluding the explanation of ancestral polymorphism due to incomplete lineage sorting, populations would have dispersed over long distances after an old lineage diversification. Evidence for multiclade presence was obtained previously by Vidal et al. for the localities of Las Cascadas and Los Muermos, close to Estaquillas [24].

Several clades in the South of the mainland predate the LGM and show a restricted distribution, suggesting a “relict population” with low rates of expansion. For example, clades S1 and S2B2, which are 187,000 and 305,000 ya respectively, are restricted to a small region of the southern coast. Clade S2A, which diverged approximately 300,000 ya, has a broader distribution but is restricted to the Western Andes, suggesting that some *L. pictus* phylogroups persisted within the limits of the LGM ice shield. Other clades include haplotypes from both Andean and coastal regions (S2B3 and S2B4). However, each of these clades harbors two subclades that are reciprocally monophyletic, with one each in the Andes and the Coastal range. Gene flow estimates between these areas suggest an exchange of individuals between the coast and the Andes predating the LGM. Pleistocene glaciations that occurred in southern South America are well documented [84], [85], and according to the dates of these events, both the origin and diversification of the main clades of *L. pictus* should be associated with the coldest Pleistocene glaciations approximately 700,000 ya and the last southern Patagonia glaciations 180,000 ya. *Liolaemus pictus* is one of the youngest species within the genus, and our molecular divergence estimates (Vera and colleagues, unpublished data) suggest that most of the species, both in the “Chilean group” and in species of the subgenus *Eulaemus* in Argentina [86], are older than *L. pictus*.

Although the levels of spatial structure were deeper for the mitochondrial than for both nuclear genes, the spatial trends of the genealogical relationships were similar among them and not in total conflict. The general mitochondrial pattern was strongly suggested by both nuclear genes, but the southern range showed a mixed distribution of related nuclear haplotypes and incomplete lineage sorting. Nevertheless, both nuclear genes suggest differentiation between the Northern and the Southern groups, thus corroborating the mtDNA gene tree showing the oldest split between lineages at approximately 39°S, and a more recent weaker structure among the southern populations. These kinds of differences between the patterns of nuclear and mitochondrial differentiation are expected given the larger N_e_ and longer sorting time to reach monophyly for the nuclear loci [87], [88].

### Historical demography

Our demographic analyses of *L. pictus* corroborate the hypothesis of population size decreases during Pleistocene glaciations [89], but these reductions started prior to the LGM, about 50,000–60,000 ya, shortly after the onset of the Llanquihue Glaciation approximately 70,000 ya. This decrease began at about the same time in both the Northern and Southern clades, but the magnitude of this fluctuation was larger in the southern populations. These results are concordant with the changes of distributional range inferred from niche modelling, and the discordance between past and present distributions is larger for southern than for northern areas. In turn this fact predicts the occurrence of larger areas for historical population expansion in the South, which fits with the observed spatial pattern of shared haplotypes among distant localities, the presence of secondary contact zones, and greater incomplete lineage sorting in nuclear genes in the southern portion of the *L. pictus* distribution. The more extensive ice sheets in the South produced extensive exclusion zones for animal and plant populations [90], and subsequent retreat of the glaciers with warming temperatures (∼17,000 ya) provided more favorable conditions for re-colonization by many species [91]. These improved conditions are reflected in the genetic signatures of both clades of *L. pictus* by rapid population growth after the ice sheet melted, but with much greater expansion in the southern group. This population expansion was likely associated with the rapid colonization of *Nothofagus* forests from southern refugia during the same period [9]. Conversely, the higher genetic structure observed for *L. pictus* in the northern part of its distribution is most parsimoniously explained by local population reductions followed by modest post-LGM population expansion with limited dispersal, as found previously in another lizard species [92].

### Pleistocene refugia

Although research is beginning to characterize LGM refugia in southern South America [93], [94], little is known compared to the Northern Hemisphere [15], [95], [96], [97], [98], [99]. Our results suggest that more than the two classical putative refugia (i.e. one Coastal and one Esat Andean), sustained populations of *L. pictus*. This is supported by several lines of evidence, including the presence of private haplotypes within glacial limits, several clades older than the LGM and distributed throughout the Andes, and high genetic diversity for many populations across the species distribution. On the other hand, the presence of probable ancestral haplotypes within LGM limits and signals of gene flow from the Andes to neighboring areas suggest the past occurrence of multiple source populations within the boundaries of the LGM ice shield. Considering the glacial history of the study area, our data suggests that during recurring glacial phases, and in addition to the Pacific Coastal refugia, *L. pictus* likely persisted in multiple Andean refugia, where genetic variation has accumulated and from where populations expanded during interglacial periods [100], [101]. This scenario is corroborated by ENM projections of potential past distributions of *L. pictus*, which indicate that during the LGM appropriate habitat persisted in the Andes. In general terms, ENM predicts the occurrence of refugia on Chiloé Island, in the central valley, adjacent Pacific coast, and several isolated valleys within the Andes. As such, many populations of *L. pictus* could have persisted in these areas, as isolated pockets within the presumed boundaries of the LGM ice shield. Similarly, this same scenario has also likely occurred during older glaciations.

Independent evidence corroborates the occurrence of ice free areas during the LGM within the Andes, specifically the Ñuble glacial gap (36°30′S) and the Malalcahuello Valley which might have acted as refugial areas [11], [102]. Evidence for within LGM ice shield refugia has been published for other groups. For example Xu et al. [13] proposed a refugium in the Andean locality of Termas del Amarillo (∼43°S) for the freshwater crab *Aegla alacalufi*. Similarly, Nuñez et al. [14] found evidence for two intra-LGM refugia in the frog *Eupsophus calcaratus*. *Liolaemus pictus* haplotypes collected from the Malalcahuello area (sites 8, 9) were highly divergent and fell in both Southern and Northern clades, Malalcahuello being the area where both groups overlapped. The same pattern was found in the mouse *Abrothrix longipilis*; in this case highly divergent haplotypes co-occur in the area of Malalcahuello (D'Elía and colleagues, unpublished data). In addition, Ruiz et al. [103] suggested that Malalcahuello was a refugial area for the large “monkey tail” tree *Araucaria araucana*. Future research will clarify the roles of Malalcahuello and Ñuble either as refugia for *L. pictus* or as areas of secondary contact of variants that differentiated elsewhere. We note that the repetition of the pattern for unrelated taxa seems to suggest that the region was probably a refugium.

The estimated ages for most clades suggest that their origins predate the LGM, therefore the most probable explanation for the observed distribution of the Andean clades S2A, S2B3 and S2B4 would be the long-term persistence of several refugia within the limits of the LGM, as suggested by the ENM. An alternative explanation would be that during the LGM, populations were driven into the Longitudinal Valley and later recolonized the Andean Cordillera and coastal areas. However, the latter scenario predicts less geographic structure within each clade. Some clades, such as S2B3 and S2B4, include haplotypes from both cordilleras (Coastal and Andes), but haplotypes from each cordillera tend to form reciprocally monophyletic groups within each of these clades. Additional evidence for our interpretation of persistent refugia within the LGM ice sheet comes from the geographic provenance of the inferred ancestral haplotypes of some haplogroups. Six of the 10 haplogroups in the cyt-b network were Andean in distribution and occurred in areas within the LGM ice sheet, which suggests that these phylogroups likely originated in these areas prior to the LGM. Additionally, the frequency with which Andean populations acted as sources and Coastal populations as sinks for gene flow is striking, and such patterns can only be explained by the occurrence of multiple Andean refugia during the LGM. Our estimates suggest several cases of gene flow from the Andes to non Andean areas. For clades S2B3 and S2B4, the predominant migration pattern was from the eastern (Argentinean localities in the Andes, within the ice shield boundaries) to the Chilean coastal mainland. According to these results, it is very likely that eastern Andean populations were a source of variants for Pacific coastal sink populations at similar latitudes. The recent review of Sèrsic et al. [98] based on lizard, small mammal, and plant species that share part of their ranges with *L. pictus*, suggests three kinds of refugia in southern South America, all outside the LGM ice shield boundary: 1) a northern area in the central valley, 2) a Pacific Coastal area including Chiloé Island, and 3) the eastern Andes outside the LGM. Palynological and genetic studies suggest that the eastern Andean refugia, at the latitude proposed in our study (37°S–44°S), would have been delimited by Andean glaciers to the West and the Argentinean steppe towards the East [104], [105], [106]. Overall, our results agree with these proposals. However, in addition to these previous proposals of refugia, we hypothesize the occurrence of refugia both in the western and eastern Andes and within the limits of the large LGM ice-shield boundary. These fragmented refugia would have been distributed from the northern extreme of the distribution of *L. pictus* south to approximately 41°S, and are characterized by unexpectedly high genetic endemism and diversity.

In agreement with similar findings from other *taxa*, these results constitute the first evidence for the intraglacial persistence of multiple lizard populations during the LGM. The proposal of a more complex scenario of several isolated refugia is further supported, and should replace the over-simplified scenario of a continuous LGM ice shield in the southern South America Andes. Future studies based on other co-distributed species are needed to test the proposed scenario.

### Taxonomic implications

Our phylogeographic patterns do not corroborate the current taxonomic arrangement within *L. pictus*, in particular in relation to most of the recognized subspecies [76]. The haplotypes gathered from specimens previously identified on the basis of their geographic origin as *L. pictus argentinus* were recovered in three well-supported clades. Our extensive sampling shows that haplotypes from specimens collected within the distributional range of *L. pictus chiloensis* fail to form a monophyletic group; two haplotypes belong to an otherwise mainland clade, but most of the individuals from most of Chiloé Island were recovered in a well supported clade, which should be considered as a different evolutionary unit. Remarkably, Chiloean specimens are morphologically distinct from those collected on the mainland. The nature of the incongruence between the mitochondrial and morphological patterns of geographic variation is for the moment unknown [37], and we have no clear evidence to support the existence of any of the previously proposed southern subspecies of *L. pictus*. However, the deep divergence (ca. 11%) between the Northern and the Southern clades suggest differences that should be recognized at the species level. The analysis of unlinked loci and morphological characters is necessary to test the mitochondrial based hypothesis [107], given that gene trees do not always correctly track species trees [108]. Although nuclear loci do not recover reciprocally monophyletic Northern and Southern groups, they tend to do so. Given that expected coalescent times are larger for nuclear loci [109], this is not an unexpected pattern. Our preliminary observations suggest considerable morphological differences, especially related to color patterns, and these seem to be concordant with the distributions of the two main clades. If correct, the Northern clade should be elevated to the species level with the name *Liolaemus septentrionalis*, and the name *L. pictus* should be restricted to the Southern group.

## Supporting Information

Table S1 **Collection localities for** ***Liolaemus pictus,*** **sample sizes (N) and subspecies to which are allocated.** Localities were grouped into four geographic zones (West Andes Cordillera: WAC; East Andes Cordillera: EAC; Coastal Cordillera: CC; and Insular Chiloé: IC, as well as into two genealogical based groups (South and North).
(DOC)

Table S2 **Haplotypes composition for each sample site for the mitochondrial region cyt-b and the nuclear genes LDB5B and EXPH5 in** ***Liolaemus pictus*** **.** Bold numbers are haplotypes distributed in more than one locality. Geographic coordinates and relative distribution of each locality are detailed in Table S1 and Fig. 1.
(DOC)

Table S3 **Analysis of molecular variance (AMOVA), for the two main haplogroups in** ***Liolaemus pictus*** **.** Each group in the AMOVA corresponds to the deepest splited clades, called northern and southern. Northern phylogroup is distributed from the northern extreme to approximately 37°S, and the Southern phylogroup is from this latitude up the southern extreme of the distribution of *L. pictus*.
(DOC)

Table S4 **Estimates of evolutionary divergence (p-distance bellow the diagonal), over sequence pairs between phylogroups (clades) in** ***Liolaemus pictus*** **.** Greyscale according to evolutionary divergence values. Values on the diagonal are intra clades p-distances. Standard error estimates are shown above the diagonal. Phylogroups codes as in Fig. 2.
(DOC)

Figure S1 **Haplotype networks for the nuclear gene EXPH5 in** ***L. pictus*** **.** Grey haplotypes correspond to the outgroups *L. lemniscatus* (L.l), and *L. tenuis* (L. t). Color codes as in the map of Fig. 1.
(TIF)
