# Supplementary material for: Lizards on Ice: Evidence for Multiple Refugia in Liolaemus pictus (Liolaemidae) during the Last Glacial Maximum in the Southern Andean Beech Forests
Source: PLoS One. 2012 Nov 27;7(11):e48358. doi: 10.1371/journal.pone.0048358 (PMC3507886; doi:10.1371/journal.pone.0048358)
Supplement: Table S1 — Collection localities for Liolaemus pictus, sample sizes (N) and subspecies to which are allocated. Localities were grouped into four geographic zones (West Andes Cordillera: WAC; East Andes Cordillera: EAC; Coastal Cordillera: CC; and Insular Chiloé: IC, as well as into two genealogical based groups (South and North). (DOC) [file pone.0048358.s001.doc]

**Table S1: Collection localities for *Liolaemus pictus,* sample sizes (N) and subspecies to which are allocated.** Localities were grouped into four geographic zones (West Andes Cordillera: WAC; East Andes Cordillera: EAC; Coastal Cordillera: CC; and Insular Chiloé: IC, as well as into two genealogical based groups (South and North).
