# Supplementary material for: Lizards on Ice: Evidence for Multiple Refugia in Liolaemus pictus (Liolaemidae) during the Last Glacial Maximum in the Southern Andean Beech Forests
Source: PLoS One. 2012 Nov 27;7(11):e48358. doi: 10.1371/journal.pone.0048358 (PMC3507886; doi:10.1371/journal.pone.0048358)
Supplement: Table S2 — Haplotypes composition for each sample site for the mitochondrial region cyt-b and the nuclear genes LDB5B and EXPH5 in Liolaemus pictus . Bold numbers are haplotypes distributed in more than one locality. Geographic coordinates and relative distribution of each locality are detailed in Table S1 and Fig. 1. (DOC) [file pone.0048358.s002.doc]

**Table S2. Haplotipes composition for each sample site for the mythochondrial region cyt-b and the nuclear genes LDB5B and EXPH5 in *Liolaemus pictus*.** Bold numbers are haplotypes distributed in more than one locality. Geographic coordinates and relative distribution of each locality are detailed in Table S1 and Fig. 1.

| **N Site** | **Locality** | ***Cytb* haplotypes** | ***LDB5B* alleles** | ***EXPH5* alleles** |
| --- | --- | --- | --- | --- |
| **1** | Valle Las Trancas | 102, 103, 104, 105 | B18 | E17, E18 |
| **2** | Parque Nahuelbuta | 112, 113 | B1, **B2** |  |
| **3** | Puren | 100 | B8 |  |
| **4** | Puren | 101 |  |  |
| **5** | R.N.Malleco Las Mentas | 114, 115, 116, 117, **118**, 119, 120, 121 |  |  |
| **6** | Lonquimay | 126 | **B2**, B7 |  |
| **7** | Lonquimay | **118**, 127, 128, 129 | **B6** | **E11**, **E1** |
| **8** | R.N. Malalcahuello (Salto El indio) | 122, 123, 124, 125 | B14, B15, B16 | **E11**, **E1**, **E7** |
| **9** | R.N. Malalcahuello | 93 | B17 | E15, E16 |
| **10** | Ref.Vn.Llaima | 106, 107, 108, 109, 110, 111 | B4 | **E1**, E2, **E7** |
| **11** | P.N Huerquehue | **36**, 38 |  |  |
| **12** | Villarrica | 40 |  |  |
| **13** | Villarrica | 39 | **B5** |  |
| **14** | Camino Parque Oncol | 77, 78, 79, 80, 81, 82 | B3 | **E8**, **E3** |
| **15** | Tres chiflones altos | 97, 98, 99 |  | **E4** |
| **16** | Cam. Hueicolla. MN Alerce costero | 88, 89, 90, 91,92 | **B6** | E9, E10, **E3** |
| **17** | Pasado Los Llolles | **36**, 37 |  |  |
| **18** | L.Ranco cca. Llifen | 33 |  |  |
| **19** | Cam.Lago Maihue | 34, 35 | **B6** |  |
| **20** | Camino La Unión a Hueicolla | 83, 84, 85, 86, 87 |  |  |
| **21** | Maicolpué | 41, 42, 43, 44, 45 | **B6** | E12, **E3** |
| **22** | Puyehue | **25**, **26**, **29** |  |  |
| **23** | Estaquillas | 15, 16, 17 |  |  |
| **24** | Estaquillas | 18, 19 ,20 ,21 ,22, 23, 24 | **B5** | **E3**, **E4** |
| **25** | Parque Alerce Andino | **46**, **47** |  |  |
| **26** | Parque Alerce Andino | **46, 47**, 48, 49, 50 |  | **E4** |
| **27** | Cuenca Río Puelo | **74** |  |  |
| **28** | Chaitén | **69** |  |  |
| **29** | Chaitén | **69** |  |  |
| **30** | Canal Garrado | **69** |  |  |
| **31** | Camino a Hua Hum | **70**, 71 |  |  |
| **32** | Villa Angostura | **70**, 76 | **B6** |  |
| **33** | H.Puyehue-Ag.Caltes | 30, 31, 32 |  |  |
| **34** | Antillanca | **25**, **26**, 27, 28, **29** |  | E5, E6 |
| **35** | Bariloche | 72, 73, **74**, 75 |  |  |
| **36** | Sur de Ancud | 1,**2,3**,4,5, |  |  |
| **37** | Sur de Ancud | **2**, **3**, 7, 8, 9, 10 |  |  |
| **38** | Sur de Ancud | **2**, 6 |  |  |
| **39** | Belben | **2**, 11, **12**, 13, 14 |  |  |
| **40** | Alto de Tantauco | **2, 12**, 51, 52, 53, 54 | B9 | E13, **E8** |
| **41** | Alto de Tantauco | 55, 56, 57, 58, 59, 60 | B10, B11 | **E8** |
| **42** | Pastahué | 62, 63, 64, 65, 66 | B12, **B6** | **E4** |
| **43** | Lago Cucao | 67, 68 | **B5** |  |
| **44** | Lago Huillinco | 61 |  | E14, **E4** |
| **45** | Parque Tantauco | 94, 95, 96 |  |  |
| **46** | Aluminé | 130 |  |  |
