# Supplementary material for: Lizards on Ice: Evidence for Multiple Refugia in Liolaemus pictus (Liolaemidae) during the Last Glacial Maximum in the Southern Andean Beech Forests
Source: PLoS One. 2012 Nov 27;7(11):e48358. doi: 10.1371/journal.pone.0048358 (PMC3507886; doi:10.1371/journal.pone.0048358)
Supplement: Table S3 — Analysis of molecular variance (AMOVA), for the two main haplogroups in Liolaemus pictus . Each group in the AMOVA corresponds to the deepest splited clades, called northern and southern. Northern phylogroup is distributed from the northern extreme to approximately 37°S, and the Southern phylogroup is from this latitude up the southern extreme of the distribution of L. pictus. (DOC) [file pone.0048358.s003.doc]

**Table S3. Molecular variance analysis (AMOVA), for the two main haplogroups in *Liolaemus pictus*.** Each group in the AMOVA corresponds to the deepest splited clades, called northern and southern. Northern phylogroup is distributed from the northern extreme to approximately 37^o^S, and Southern clade is from this latitude up the southern extreme of the distribution of *L. pictus*.

| **Source of variation** | **d.f.** | **Sum of Squares** | **% of variation** |
| --- | --- | --- | --- |
| Among groups | 1 |  |  |
| (Northern and Southern) |  | 1201.38 | 55.50 |
| Among populations |  | 2024.48 | 33.88 |
| within groups | 44 |  |  |
| Within populations | 150 | 486.00 | 10.62 |
