# Supplementary material for: Lizards on Ice: Evidence for Multiple Refugia in Liolaemus pictus (Liolaemidae) during the Last Glacial Maximum in the Southern Andean Beech Forests
Source: PLoS One. 2012 Nov 27;7(11):e48358. doi: 10.1371/journal.pone.0048358 (PMC3507886; doi:10.1371/journal.pone.0048358)
Supplement: Table S4 — Estimates of evolutionary divergence (p-distance bellow the diagonal), over sequence pairs between phylogroups (clades) in Liolaemus pictus . Greyscale according to evolutionary divergence values. Values on the diagonal are intra clades p-distances. Standard error estimates are shown above the diagonal. Phylogroups codes as in Fig. 2. (DOC) [file pone.0048358.s004.doc]

**Table S4.** **Estimates of evolutionary divergence (p-distance bellow the diagonal), over sequence pairs between phylogroups (clades) in *Liolaemus pictus*.** Greyscale according to evolutionary divergence values. Values on the diagonal are intra clades p-distances. Standard error estimates are shown above the diagonal. Phylogroups codes as in Fig. 2.
