# Supplementary figures and images for: Lizards on Ice: Evidence for Multiple Refugia in Liolaemus pictus (Liolaemidae) during the Last Glacial Maximum in the Southern Andean Beech Forests
Source: PLoS One. 2012 Nov 27;7(11):e48358. doi: 10.1371/journal.pone.0048358 (PMC3507886; doi:10.1371/journal.pone.0048358)

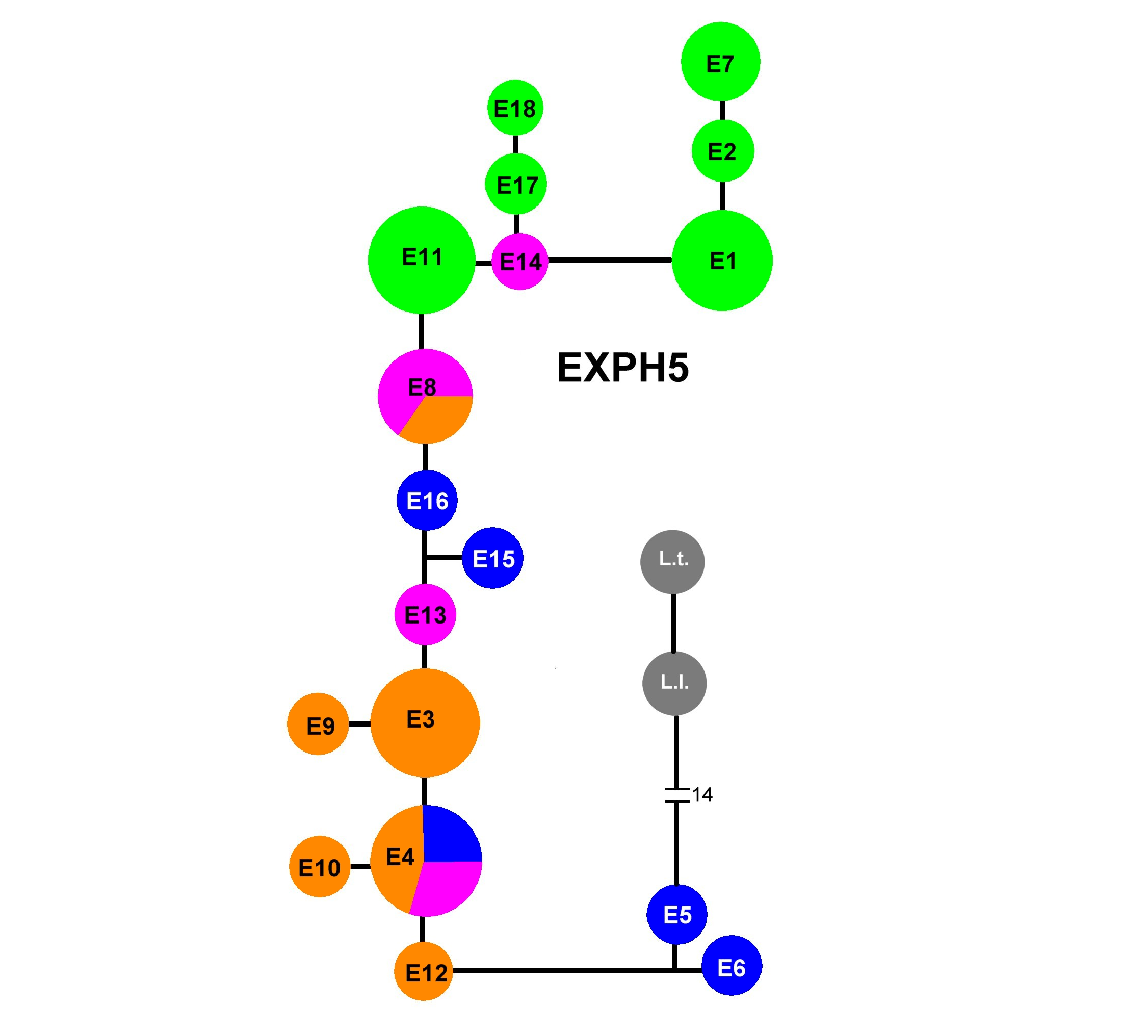

Supplement: Figure S1 — Haplotype networks for the nuclear gene EXPH5 in L. pictus . Grey haplotypes correspond to the outgroups L. lemniscatus (L.l), and L. tenuis (L. t). Color codes as in the map of Fig. 1. (TIF) [file pone.0048358.s005.tif]
